# Grass species with smoke‐released seed dormancy: A response to climate and fire regime but not photosynthetic pathway

**DOI:** 10.1111/plb.13479

**Published:** 2022-11-06

**Authors:** B. B. Lamont

**Affiliations:** ^1^ Ecology Section, School of Molecular and Life Sciences Curtin University Perth WA Australia

**Keywords:** C3, C4, grasses, mediterranean, photosynthetic pathways, savanna, seed germination

## Abstract

Both C3 and C4 photosynthetic pathways and smoke‐released seed dormancy occur among grasses. C4 species evolved from C3 species as seasonality and fire frequency increased and might therefore imply that their smoke sensitivity increased. I searched the worldwide literature for reports on germination responses among grasses, whose photosynthetic pathway was known, to treatment by smoke. Data were obtained for 217 species and 126 genera. While subfamilies tended to be C3 (Pooideae), C4 (Chloridoideae) or a mixture (Panicoideae), a beneficial smoke response was independent of their photosynthetic pathway. The only exceptions were Danthonioideae (C3, non‐smoke responsive) and *Triodia* (C4, smoke responsive). One third of both C3 and C4 genera were smoke responsive. Even within genera, 90% of species showed contrasting smoke responses, confirming that smoke sensitivity is rarely taxonomically constrained. Data on photosynthetic pathway, climate, fire regime and vegetation were compiled for 15 regions that formed four distinct groups: 1) In warm climates with aseasonal rainfall, C4 grasses are moderately better represented, with crown fires and limited smoke responses. 2) In cool regions, most species are C3, with surface‐crown fires and lack smoke responses. 3) In warm regions with summer rain (savannas), most species are C4, with surface fires and lack smoke responses. 4) In Mediterranean‐climate regions with summer drought, most species are C3, with crown fires and smoke‐released dormancy. Thus, even though C3 and C4 grasses are equally capable of expressing smoke sensitivity, their response depends on the region’s climate and fire regime that also dictate which photosynthetic pathway dominates.

## Graphical abstract

Among worldwide grass species, four types of smoke‐assisted seed dormancy release can be recognized, based on % with C4 photosynthesis, vegetation type, rainfall seasonality, and type and frequency of fire.

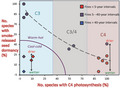

**Highlights**

- On a world scale, smoke‐released seed dormancy exists equally among 40% of C3 and C4 grass species and one‐third of genera and is not taxonomically constrained.
- Some floras have only C3 grasses (summer dry) and some only C4 (summer wet) and most are dominated by one or the other.
- Smoke sensitivity and photosynthetic pathway are readily divided into four regional types based on climate and fire regime.
- Smoke sensitivity is poorly represented in frequently‐burnt C4 grassland/savannas but is almost universal in intensely‐burnt C3 mediterranean shrubland/forests.
- Moderate levels of smoke sensitivity among C3/C4 grasses under intermediate climates confirm that the presence/absence of smoke sensitivity elsewhere is unrelated to photosynthetic pathway.

## INTRODUCTION

Grasslands with C4 began to replace the C3 grasslands in Africa 14–16 million years ago (Ma), with a marked expansion at 4–9 Ma, which has been attributed to the onset of many environmental changes, especially the onset of summer rain and more frequent fires (Retallack 1992; Jacobs *et al*. 1999; Kürschner *et al*. 2008; Osborne 2008; Scheiter *et al*. 2012). C4‐type photosynthesis has arisen at least 20 times among tropical grasses (family Poaceae) throughout the world (Linder *et al*. 2018), and it is now widespread at both the subfamily and generic levels (Edwards & Smith 2010). Subsequent reviews either make no reference to fire in the conversion of C3 to C4 grasses (Linder *et al*. 2018) or only mention fire in relation to resprouting (Simpson *et al*. 2021). None mentions fire effects on germination, although it is well‐established that dormancy release among many grasses is promoted by smoke (Ghebrehiwot *et al*. 2012; Schwilk & Zavala 2012; Carthey *et al*. 2018).

Seed dormancy is expected to be most adaptive in highly seasonal climates, where seedling recruitment is only likely to succeed once the soil is moist and moderately warm. In fire‐prone habitats, optimal recruitment conditions are favoured by the post‐fire environment when large bare patches, with increased levels of light, moisture and nutrients, are created. Thus soil‐stored seeds will have an adaptive advantage if their inherent dormancy is broken by smoke released from fire that heralds optimal recruitment conditions (Pausas & Lamont 2022). Environmentally induced dormancy will then be maintained until the onset of the first substantial rains at mild temperatures. This imposed dormancy period may be short‐circuited if the smoke chemicals are not absorbed until dissolved in soil water associated with the wet season. Once absorbed, the karrikins link with dedicated proteins to produce hydrolytic enzymes that degrade stored carbohydrates and proteins, which enable germination to commence once hydrothermal conditions are suitable (Lamont *et al*. 2019). There is some evidence that smoke chemicals may also hasten the rate of germination, as distinct from their effect on dormancy release (Hodges *et al*. 2021), which might be especially adaptive under summer‐rainfall climates with their less effective rain.

This raises the question as to whether the germination of C4 grasses is therefore more likely to be promoted by smoke chemicals than that of C3 grasses. Is this another trait, like resprouting (Paula & Pausas 2011), that gives C4 grasses a competitive edge in frequently burnt savannas but that has been ignored? However, current evidence actually shows smoke‐released dormancy is poorly represented among some C4 grasses, and the reverse may be true. The possible explanations are numerous, including fires are so frequent that seed dormancy is no longer adaptive (Pausas & Lamont 2022). C3 grasses are widespread in uniform rainfall, cool to temperate climates that may or may not be highly fire‐prone (Tsuyuzaki & Miyoshi 2009). In particular, they are also characteristic of highly seasonal, fire‐prone mediterranean climate regions, where smoke‐released dormancy is also expected to be well represented (Roche *et al*. 1997; Reyes & Trabaud 2009). To resolve these conflicting expectations on the relationship between photosynthesis type and smoke responsiveness, I collated data from the literature on the effect of smoke on germination of C3 and C4 species located throughout the world and show that the answer is almost entirely context‐driven (climate plus the associated fire regime), rather than dependent on their photosynthetic pathway or taxonomic affinity.

## METHODS

I fed the keywords ‘grass’, ‘smoke’, ‘seed’ and ‘dormancy’ into Google^®^ and Google Scholar^®^. Papers were selected that met the criteria of: (i) >3 grass species tested for the effect of smoke on breaking dormancy *versus* controls including their statistical significance; (ii) covered most fire‐prone regions of the world and included data on climate, fire regime and vegetation type; and (iii) could be downloaded from the web or were available through the Curtin University library. Since most papers used smoke water, there was no reason to suspect a bias in the type of smoke application between C3 and C4 species, and the focus was on presence/absence of a smoke response rather than its quantitative effect. No distinction was made between tests using dry smoke, smoke water or karrikins. This yielded 40 suitable papers that are cited in the reference list. Results for each species were placed in the categories ‘plus’ (smoke beneficial), ‘nil’ or ‘minus’ (smoke inhibitory) based on statistical tests in those papers. I then searched the web for whether the photosynthesis type of each species was known. Suitable data were available for 217 species and 126 genera. The subfamily for each species was noted. I also tabulated the percentage germination of controls and smoke treatment, if provided, and whether considered statistically significant. Suitable data were available for 145 species in 61 genera. The data were collated into 14 regions based on climate, fire‐proneness and vegetation type. The raw data are given in Table S1.

To test whether smoke responses were not constrained by taxonomic affinity or photosynthesis type, I undertook four tasks. Species were allocated to their smoke response (positive *versus* nil or negative response) within their subfamily that was either C3 (Pooideae), C4 (Chloridoideae) or mixed (Panicoideae), with the 32 remaining species placed in a miscellaneous group of five families, to see if smoke responses were unaffected by subfamily/photosynthesis type. Since there were seven C3 and 81 C4 species in Panicoideae, this provided an opportunity to test if they were allocated equally between the two smoke‐response types as a mark of their independence within a subfamily. To test for independence at the within‐genus level, whether the smoke response was the same or mixed for those with two or three or more species was collated. To ensure possible bias introduced by varying number of species per genus was removed, each genus was represented by a value of one if all its species had the same smoke response, and 0.5 in each category if they differed. Where actual germination data were compared, the means of the smoke response and control for all species in the genus were calculated so that each genus contributed only one value to each category in the analysis.

The categorical data were submitted to Yates‐corrected Chi‐square or Fisher's Exact tests, depending on class sizes (VassarStats, 2012–21©). Tests were two‐tailed when there was no *a priori* expectation of a difference between the smoke‐response types, and one‐tailed on the expectation that smoke would promote germination over the controls. Since these analyses showed that smoke‐released seed dormancy is spread widely and equally across the two photosynthesis types at taxonomic ranks from subfamilies to subgenera, this indicated that, even when there were taxonomic constraints on photosynthesis type, there were no photosynthesis‐type constraints on the smoke response. The smoke response of each species was therefore treated as an independent data point in subsequent analyses. This approach was also supported by the species occurring in widely disparate geographic locations, minimizing opportunities for spatial autocorrelation of taxonomically related species (except perhaps for *Triodia*). The 2 × 2 quantitative (germination) data could not be analysed by conventional two‐way ANOVA as the smoke–control pairings created a third variable, a species effect, so were not independent. Thus, a non‐paired *t*‐test was conducted on the C3 *versus* C4 data per genus, and a paired *t*‐test was conducted on the smoke *versus* control data per genus.

When relative number of species in the region with a smoke response was regressed against relative number with C4‐type photosynthesis, the data formed four highly disparate groups: groups 1/3 were separated from 2/4 by at least a 25% difference in percentage C4, and group 1 was separated from groups 2–4 by at least 25% smoke‐responsiveness, making more formal ordination approaches to identify groups unnecessary. Data for the Restionaceae in the Cape province of South Africa were added as one dataset among the 15 collated by region at this stage as: (i) no grass data were available from this region, (ii) it is sister to the Poaceae and its growth form and habitats are matched, and (iii) this clade is well studied for its smoke requirements (He *et al*. 2016). The means ± confidence intervals at 95% of these groups were then calculated for comparative purposes. The common climate, fire regime and vegetation types for each group were obtained from the relevant papers and placed in categories for comparative purposes.

## RESULTS

Ignoring their geographic distribution, the germination of 39.2% of 217 worldwide grass species benefitted from smoke (Table 1A). While the best‐represented subfamilies were either C3 (Pooideae), C4 (Chloridoideae) or mixed (Panicoideae), their smoke responses were matched (41–45% of species). The only exception appears to be Danthonioideae, whose ten species were all C3 and non‐smoke‐responsive (Table S1). Within the mixed subfamily (Panicoideae), there was no difference in smoke response between C3 (43%) and C4 (41%) species (Table 1B). Within genera, for half of those represented by two species, half had one smoke‐responsive and one non‐smoke‐responsive species; of those with three or more species, almost 90% were mixed smoke and non‐smoke responsive (Table 1C). The only exception appears to be *Triodia* whose seven species were C4 (or CAM) and smoke‐responsive (Table S1). Removing any bias caused by varying number of species per genus on the results by representing each genus by one type or another (or 0.5 to each if mixed) resulted in identical smoke responses between C3 (34%) and C4 (33%) (Table 1D). Overall, on a per genus basis, C3 species germinated ~13% better than C4 species, for both controls and smoke‐treated (*P* = 0.014, two‐tailed) (Table 1E). Smoke improved germination of both C3 and C4 genera by ~8% (*P* < 0.001, one‐tailed). The smoke‐treated C3 species germinated 21% more than the control C4 species.

**Table 1:** Collated results for the total dataset, taking photosynthesis type into account in relation to smoke responses. (A) number of species in each subfamily, (B) allocations within Panicoideae (the only subfamily with both C3 and C4), (C) number of genera with two or more species assessed with the same or differing response to smoke, (D) number of genera allocated to each photosynthesis type and smoke response type (multiple species in each genus represented by one value), (E) actual percentage germination per genus. Data collated from Table S1.

(A) Number of species in each subfamily recorded in this study for their germination response to smoke. Beneficial = positive/total. (Total of 217 species assessed)			
subfamily	positive smoke response	no or negative response	smoke beneficial (%)
Panicoideae (C3/4)	36	52	40.9
Chloridoideae (C4)	15	18	45.4
Pooideae (C3)	27	37	42.2
Remaining 5 (C3 or C4)	7	25	22.6
Allocation (%)	39.2	60.8	
Chi‐square = 4.92, 3 df, *P* = 0.1777 (two‐tailed)			

(B) Number of species in the subfamily Panicoideae that are C3/C4 *versus* their germination response to smoke.			
Panicoideae	positive smoke response	no or negative response	smoke beneficial (%)
C3	3	4	42.9
C4	33	48	40.7
Fisher exact probability test: *P* = 1.000 (two‐tailed)			

(C) Number of genera with two or more species assessed with the same or differing response to smoke of two or more species assessed.		
smoke response between species	two species assessed per genus	three or more species assessed per genus
Same	6	3
Different	6	19
Differ (%)	50.0	86.4

(D) Number of genera (if contrasting values, then 0.5 allocated to each category) per category of germination response to smoke for a total of 126 genera, 2 × 2 contingency analysis. Beneficial = positive/total.			
photosynthesis type	positive smoke response	no or negative response	beneficial (%)
C3	21	41	34.4
C4	21	43	32.8
Allocation (%)	33.3	66.7	
*P* (Yates Chi‐square): 1.000 (two‐tailed)			

(E) Mean ± 95% confidence interval (%) for effect of smoke on germination of 30 C3 genera and 31 C4 genera. Means used if multiple species in genus. Non‐paired *t*‐test with no hypothesis (two‐tailed) for C3 *versus* C4, paired *t*‐test with hypothesis smoke > controls (one‐tailed).								
photosynthesis type	smoke response	control	mean	variable	test	*P*		
C3	65.2 ± 10.9	55.0 ± 11.6	60.1%	C3/C4:	*t* = 2.5, 120 df, two‐tailed	0.0138	57	10%
C4	50.6 ± 12.5	44.2 ± 9.3	47.4%	Smoke response:	*t* (paired) = 3.47, 60 df, one‐tailed	0.0005	46.7	
Mean	57.8%	49.4%						

That global analyses as described above obscure patterns revealed at other scales become clear when the datasets per region are compared (Table 2). Five regions have no C4 grasses, and three regions have only C4 grasses, with an average of 46.7% of grasses possessing C4 across all regions. One region has only smoke‐responsive grasses (SW Australia) – although the sample is small –, and one region has no smoke‐responsive grasses (Cerrado in Brazil), with an average of 33.9% positively smoke‐responsive on a regional basis. When inhibition cases are considered, this drops to a mean of 27.0%. Overall, the data are readily divisible into four groups based on climate, vegetation type, fire regime, the relative incidence of C4 and smoke‐responsive grasses.

**Table 2:** Number of Poaceae species with germination stimulated (plus), unaffected (zero) or inhibited (minus) by smoke treatment. Number of species with C4‐type photosynthesis (rather than C3) used to rank sites from 100% to 0%. Note that the Restionaceae (‘grass’) was used for the Cape province, South Africa, as no data were available for species in its sister family, Poaceae, in this mediterranean region. The four shaded bands represent the four response groups recognized in Figs. 1 and 2. uni(sum) = uniform (summer) = minor rainfall maximum, surf = surface (fire type), mod = moderate.

site	group	C4	plus (+)	zero	minus (−)	plus (%)	(+ − ‐)/total	wet season	rainfall^1^	temperature^2^	vegetation	fire type	fire intervals^3^	references
Shrubland/woodland, SW Australia	1	0.0	5	0	0	100.0	100.0	winter	moderate	warm	shrub‐tree	crown	3	Dixon *et al*. (1995); Roche & Dixon (1997); Roche *et al*. (1998); Smith *et al*. (1999); Enright & Kintrup (2001)
Shrublands/wetlands, Cape, South Africa	1	0.0	161	29		84.7	84.7	winter	mod/high	warm	‘grass’‐shrub	crown	3	He *et al*. (2016)
Shrubland, Mediterranean Basin	1	0.0	8	2	0	80.0	80.0	winter	moderate	warm	shrub‐tree	crown	3	Adkins & Peters (2001); Pérez‐Fernández & Rodríguez‐Echeverría (2003); Stevens *et al*. (2007); Reyes & Trabaud (2009); Long *et al*. (2011);
Sagebrush steppe, Central‐W USA	4	0.0	2	4	3	22.2	−11.1	uniform	moderate	cool	shrub	crown	3	Blank & Young (1998); Ghebrehiwot *et al*. (2009)
Shrubland, N Japan	4	0.0	0	4	2^#^	0.0	−33.3	uniform	moderate	cool	shrubland	crown	3	Tsuyuzaki & Miyoshi (2009)
Shortgrass prairie, NW USA	4	9.1	1	5	0	16.7	16.7	summer	moderate	cool	grass	surface	1	Ely (2016)
Prairie, W Canada	4	11.1	2	7	0	22.2	22.2	summer	low	cold	grass	surface	1	Abu *et al*. (2016); Yao *et al*. (2017)
Grassy woodland, SE Australia	2	43.5	8	13	2	33.3	26.1	uni(sum)	moderate	warm	grass‐tree	surf/crown	2	Clarke *et al*. (2000); Tsuyuzaki & Miyoshi (2009); Carthey *et al*. (2018)
Eucalypt forest, SE Australia	2	60.0	8	11	1	40.0	35.0	uni(sum)	high	warm	shrub‐tree	crown	2	Read & Bellairs (1999); Penman *et al*. (2008)
Cerrado grasslands, Central Brazil	3	90.0	0	11	0	0.0	0.0	summer	moderate	hot	grass‐shrub	surf/crown	1	Ramos *et al*. (2019); Gorgone‐Barbosa *et al*. (2020);
Tallgrass prairie, Central USA	3	90.9	2	8	0	20.0	20.0	summer	moderate	warm	grass	surface	1	Jefferson *et al*. (2008); Chou *et al*. (2012); Schwilk & Zavala (2012)
Grassland, South Africa	3	94.7	3.5*	15.5	0	18.4	15.8	summer	moderate	warm	grass	surface	1	Ghebrehiwot *et al*. (2012)
Semiarid savanna, NW Australia	3	100.0	8	12	2	36.4	27.3	summer	low	hot	grass‐shrub	surf/crown	2	Erickson (2015)
Savanna, North Africa	3	100.0	1	7	0	12.5	12.5	summer	high	hot	grass‐tree	surface	1	Dayamba *et al*. (2008); Dayamba *et al*. (2010)
Tropical savanna, N, NE Australia	3	100.0	5	14	3	22.7	9.1	summer	mod/high	hot	grass‐shrub	surface	1	Tang *et al*. (2003); Williams *et al*. (2005); Scott *et al*. (2010); Gamage *et al*. (2014)

*0.5 allocated when two studies gave opposing results. ^1^Low = <250 mm year^−1^, moderate = 250 mm year^−1^, high = >750 mm year^−1^. ^2^Cold = mean annual <10 °C, warm = 10–25 °C, hot = >25 °C. ^3^1 = mean <5 years, 2 = 5–40 years, 3 = >40 years. ^#^3 Cyperaceae, sister to Poaceae, added as only three Poaceae species available.

**Fig. 1:**
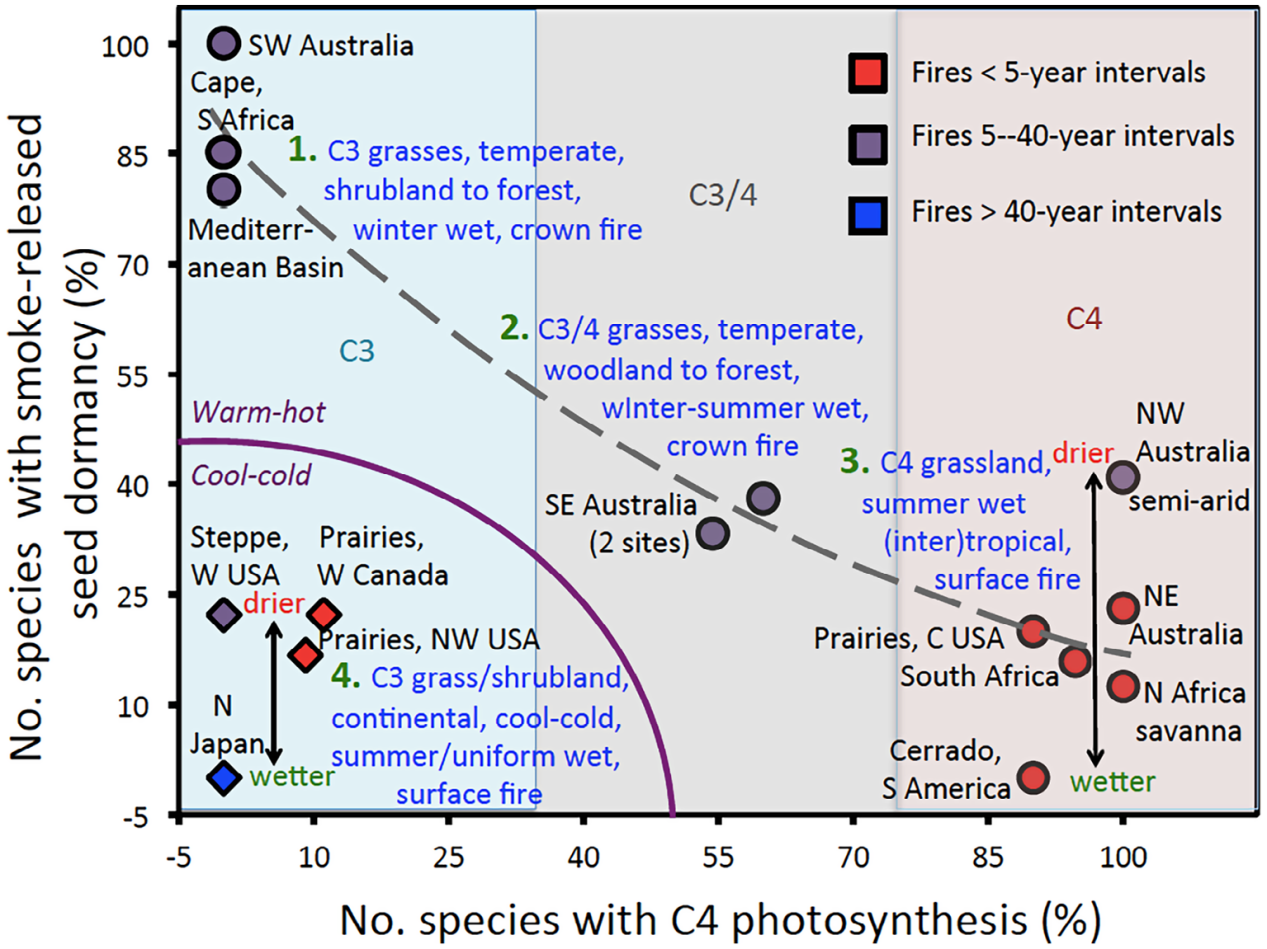
Relative number of grass species with smoke‐assisted seed dormancy breakage in 15 regions, as described in Table 1, *versus* relative number of grass species in those studies with C4‐type photosynthesis. Four types of response can be recognized, based on percentage C4 species, vegetation type, rainfall seasonality and type and frequency of fire. An exponential‐type curve has been fitted to the 11 sets of studies in warm regions (circles) omitting the four C3‐dominated floras in the cool‐cold regions (diamonds). The regions dominated by C3, C4 or mixed grasses are indicated by the coloured columns.

The first group is composed solely of C3 grasses (or more generally graminoids), with 75–100% smoke‐responsive in mediterranean (summer‐dry) shrublands to forests with moderately frequent crown fires (Fig. 1). The second group are essentially C3 with 0% (wetter)‐25% (drier) smoke‐responsive in grasslands/shrublands in cool‐cold, summer‐wet to aseasonal climates with surface fires, sometimes almost fire‐free. The third group are essentially C4 grasses with 0% (wetter)‐40% (drier) smoke‐responsive in grasslands, summer‐wet often frequent surface fires except in semiarid regions. The fourth group is intermediate, with a mixture of C3/C4 species, grassy woodland to forests with uniform rain and moderately frequent crown fires. In groups 3 and 4, there was a gradient from relatively wet to dry in terms of increasing abundance of smoke‐responsive species. This was most notable in group 3 with the high‐rainfall, Cerrado grasslands lacking any smoke‐responsive species (90% C4) to semiarid NW Australia with 40% of grasses being smoke‐responsive species (100% C4). This pattern was not evident among the three mediterranean regions.

**Fig. 2:**
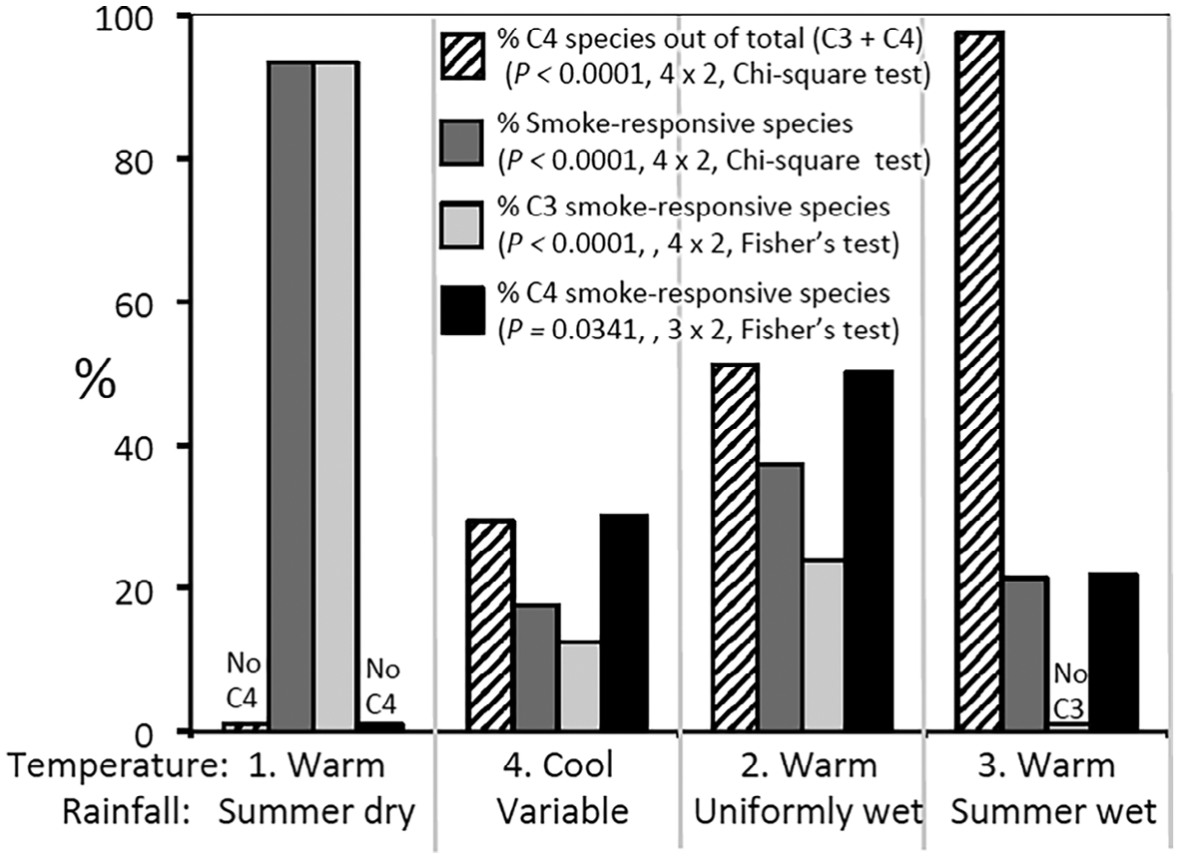
Allocation of C3, C4 and smoke‐responsive species to the four groups recognized in Table 2 and Fig. 1, except that Restionaceae data were omitted from group 1. Contingency table analyses given are based on raw data for presence or absence in each category. Temperature = mean annual temperature class (see Table 2 notes). Note the rise in C4 species representation (first bar) across the four groups but no gradient pattern for the other three attributes assessed.

The trends become clearer when the data for the various regions in each group are collated (Fig. 2). When they are arranged to give an increase in C4 species representation across the four groups, from 0% to almost 100%, equivalent gradient patterns for the other three attributes only arise when the cool‐region group, with lowest incidence of smoke‐responsive species, is omitted (as in Fig. 1). Here, smoke‐responsive species in the mediterranean group (essentially C3) is 2.5 times that in the uniform‐rainfall group, with similar total rainfall (C3 and C4 equally abundant) and 4.5 times that in the summer‐rainfall group (essentially C4). All smoke‐responsive species in the mediterranean regions were C3, and this was four times the level for C3 species in the uniform‐rainfall group. Half of the C4 species in the uniform‐rainfall group were smoke‐responsive, and this was 2.5 times that in the summer‐rainfall group which was essentially C4. Thus, the percentage of C3 species that was smoke‐responsive in the mediterranean group was much higher than in the cool and uniformly wet groups, but the percentage of C4 species that was smoke‐responsive in the groups was much higher than in the summer‐wet group.

## DISCUSSION

This meta‐analysis of C3/C4 grasses *versus* smoke‐released seed dormancy at a global scale shows that there are almost no taxonomic or photosynthetic pathway constraints to the presence of smoke responsiveness by a species. At any taxonomic rank, a given species is ~40% likely to be smoke‐responsive, whether its photosynthesis type is C3 or C4 (Table 1). Thus, each species can be treated as an independent unit for statistical purposes. The major constraints instead are centred on climate and fire regime to which the incumbent species have adapted over time. Clearly, there is something about the mediterranean environment that promotes both the presence of C3 grasses and smoke‐responsiveness, and the savanna environment that promotes both the presence of C4 grasses and dearth of smoke‐responsiveness, even though both their vegetation types are highly fire‐prone. To this we can add: there is something about intermediate and cool climates that promotes intermediate levels of both types.

The stimulus for germination of most species, here C4 grasses, proved not usually to be caused by smoke under grassy savanna climates (with frequent surface fires). For example, the seeds (caryopses) of many grass species in the Cerrado have a large non‐dormant component (Fontenele *et al*. 2020). However, it would be misleading to interpret this to mean that the seeds of C4 grasses are not fire‐adapted. Thus, seeds of three of five C4 grasses from Brazilian grasslands germinated as well as the controls after 2 min at 90 °C (Overbeck *et al*. 2005), showing substantial heat tolerance. Cuello *et al*. (2020) recorded 21 grass seedlings in a burnt Uruguay C4 grassland, compared with just four in the unburnt sites. And non‐dormancy coupled with (a) high heat tolerance and/or (b) fire‐stimulated flowering (Fontenele *et al*. 2020) enables seeds to take advantage of exceptionally wet seasons either (a) following or (b) between fires, which stored, smoke‐responsive seeds cannot do, as they must germinate whenever fires occur, even if followed by a poor growing season.

On the other hand, most species, here C3 grasses, under mediterranean climates (with moderately frequent crown fires), store their seeds in the soil and dormancy breakage requires a fire‐related stimulus, here smoke (Lamont *et al*. 2017, 2019). Again, species in cool‐cold climates, here predominantly C3 grasses (as noted by Simpson *et al*. 2021), with a wide range of fire frequencies, may rely on factors other than smoke, such as cold stratification, to promote dormancy release (Montalvo *et al*. 2002). Fire‐prone woodlands and forests have species, here a mixture of C3 and C4 grasses, with stored and non‐stored seeds that may or may not be smoke‐responsive as recruitment may also occur inter‐fire (Carthey *et al*. 2018).

Thus, the smoke response is driven solely by the fire regime (seasonality and frequency) as controlled by the climate and its associated vegetation. No physiological interaction with the photosynthetic pathway is required as both C3 and C4 grasses can be equally smoke‐sensitive. The gradual evolution of C4 grasses out of a C3 heritage has been attributed to a plethora of factors (see Introduction), including a strong correlation between resprouting in response to frequent fire and the adoption of the C4 pathway (Simpson *et al*. 2021). But the concurrent adoption of smoke‐released seed dormancy, as another potential trait to cope with frequent fire, is not one of them. In fact, the dearth of seed storage among summer‐wet grasslands is notable (Lamont *et al*. 2017; Pausas & Lamont 2022). Meanwhile, seed dormancy and its breakage by fire‐related stimuli became almost mandatory among the C3 grasses in the developing mediterranean climate regions over the last 10 million years as the increasingly severe summer‐drought, fire‐prone environments deterred inter‐fire recruitment (Lamont & He 2017; Rundel *et al*. 2018). Note that, since the binary data required were only available for a fraction of grass taxa, the results of this meta‐analysis must be regarded as indicative rather than absolute.

## Supporting information


**Table S1** Grass species allocated to photosynthesis type (C3, C4) and smoke‐released seed dormancy (1 = positive effect, 0.5 = conflicting data, 0 = no effect, −1 = negative effect). Subfam(ilies): Pa = Panicoideae, Po = Pooideae, An = Andropogoneae, Ch = Chloridideae, Mi = Micrairoideae, Ar = Aristoideae, Eh = Ehrhartoideae, Da = Danthonioideae. Sig = statistically significant difference (no details in reference). Nomenclature follows that given in the relevant papers, with spelling errors corrected as required.
