# Supplementary material for: Grass species with smoke‐released seed dormancy: A response to climate and fire regime but not photosynthetic pathway
Source: Plant Biol (Stuttg). 2022 Nov 6;25(1):24–31. doi: 10.1111/plb.13479 (PMC10099466; doi:10.1111/plb.13479)
Supplement: Supplementary file 1 — Table S1 Grass species allocated to photosynthesis type (C3, C4) and smoke‐released seed dormancy (1 = positive effect, 0.5 = conflicting data, 0 = no effect, −1 = negative effect). Subfam(ilies): Pa = Panicoideae, Po = Pooideae, An = Andropogoneae, Ch = Chloridideae, Mi = Micrairoideae, Ar = Aristoideae, Eh = Ehrhartoideae, Da = Danthonioideae. Sig = statistically significant difference (no details in reference). Nomenclature follows that given in the relevant papers, with spelling errors corrected as required. [file PLB-25-24-s001.docx]

**7. Appendix**

**Table S1.** Grass species allocated to photosynthesis type (C3, C4) and smoke-released seed dormancy (1 = positive effect, 0.5 = conflicting data, 0 = no effect, -1 = negative effect). Subfam(ilies): Pa = Panicoideae, Po = Pooideae, An = Andropogoneae, Ch = Chloridideae, Mi = Micrairoideae, Ar = Aristoideae, Eh = Ehrhartoideae, Da = Danthonioideae. Sig = statistically significant difference (no details in reference). Nomenclature follows that given in the relevant papers, with spelling errors corrected as required.

|  | **C4 grasses** |  |  |  | **Smoke** |  | **C3 grasses** |  |  |  | **Smoke** | |
| --- | --- | --- | --- | --- | --- | --- | --- | --- | --- | --- | --- | --- |
|  |  |  |  |  | **response** |  |  |  |  |  | **response** | |
| **Genus** | **Species** | **Subfam** | **Control** | **Smoke** | **rank** | **Genus** | **Species** | **Subfam** | **Control** | **Smoke** | **rank** | |
| Digitaria | breviglumis | Pa |  | sig | 1 | Vulpia | myurus | Po |  | sig | 1 |  |
| Heteropogon | triticeus | Pa |  | sig | 1 | Bromus | tectorum | Po | 32 | 59 | 1 |  |
| Euclasta | condylotricha | Pa | 38 | 58 | 1 | Bromus | tomentellus | Po | 56 | 78 | 1 |  |
| Imperata | koenigii | Pa | 50 | 62 | 1 | Elymus | glaucus | Po | 61 | 81 | 1 |  |
| Heteropogon | contortus | Pa | 4 to 40 | 54/54 | 1 | Oplismenus | aemulus | Pa |  | sig | 1 |  |
| Hyparrhenia | hirta | An | 3 | 22 | 1 | Poa | labillardieri | Po | 10 | 24 | 1 |  |
| Panicum | maximum | Pa | 80 | 95 | 1 | Stipa | scabra subsp. | Po | 14 | 34 | 1 |  |
| Triodia | angustata | Ch | 4 | 45 | 1 | Dactylis | glomerata | Po | 51 | 90 | 1 |  |
| Triodia | basedowei | Ch | 18 | 54 | 1 | Aira | elegans | Po | 3 | 11 | 1 |  |
| Triodia | shovellana hill | Ch | 40 | 67 | 1 | Alopecurus | myosuroides | Po |  | sig | 1 |  |
| Eriachne | pulchella | Mi | 71 | 96 | 1 | Avena | fatua | Po | 39 | 87 | 1 |  |
| Triodia | brizoides | Ch | 10 | 74 | 1 | Melica | ciliata | Po | 56 | 96 | 1 |  |
| Triodia | epactia | Ch | 51 | 75 | 1 | Avena | sterilis | Po | 21.5 | 73 | 1 |  |
| Triodia | pungens | Ch | 33 | 92 | 1 | Phalaris | paradoxa | Po | 50 | 100 | 1 |  |
| Triodia | wiseana | Ch | 33 | 82 | 1 | Hordeum | leporinum | Po | 65 | 100 | 1 |  |
| Triodia | melvillei | Ch | 47 | 73 | 1 | Austrostipa | compressa | Po | 36 | 86 | 1 |  |
| Bouteloua | sp | Ch |  | sig | 1 | Neurachne | alopecuroidea | Pa | 60 | 100 | 1 |  |
| Bouteloua | gracilis | Ch | 42 | 99 | 1 | Amphipogon | amphipogonoides | Ar | 19 | 53 | 1 |  |
| Digitaria | diffusa | Pa |  | sig | 1 | Tetrarrhena | laevis | Eh | 1 | 18 | 1 |  |
| Digitaria | ramularis | Pa |  | sig | 1 | Stipa | compressa | Po | 10 | 80 | 1 |  |
| Eragrostis | cilianensis | Ch |  | sig | 1 | Bromus | ciliatus | Po | 50 | 80 | 1 |  |
| Eragrostis | leptostachya | Ch |  | sig | 1 | Achnatherum | occidentalis | Po | 20 | 60 | 1 |  |
| Eragrostis | sororia | Ch |  | sig | 1 | Achnatherum | hymenoides | Po | 15 | 45 | 1 |  |
| Panicum | effusum | Pa |  | sig | 1 | Poa | annua | Po |  | sig | 1 |  |
| Paspalidium | distans | Pa |  | sig | 1 | Avena | barbata | Po |  | sig | 1 |  |
| Dichanthium | sericeum | Pa | 67 | 90 | 1 | Stipa | rudis | Po |  | sig | 1 |  |
| Sorghum | leiocladum | Pa | 27 | 55 | 1 | Echinopogon | caespitosus | Po |  | sig | 1 |  |
| Panicum | decompositum | Pa | 8 | 63 | 1 | Echinolaena | inflexa | Po | 0 | 2 | 0 |  |
| Panicum | effusum | Pa | 0 | 17 | 1 | Ehrharta | calycina | Eh | 77 | 67 | 0 |  |
| Paspalidium | distans | Pa | 18 | 31 | 1 | Brachypodium | pinnatum | Po |  | NS | 0 |  |
| Sorghum | halepense | Pa | 12 | 100 | 1 | Elymus | sp | Po |  | NS | 0 |  |
| Panicum | virgatum | Pa | 30 | 79 | 1 | Elymus | trachycaulus | Po | 67 | 71 | 0 |  |
| Themeda | triandra | Pa | 75 | 87 | 1 | Dichanthelium | oligosanthes | Pa | 28 | 39 | 0 |  |
| Cymbopogon | refractus | Pa |  | sig | 1 | Danthonia | californica | Da | 79 | 82 | 0 |  |
| Bothriochloa | bladhii | Pa |  | sig | 1 | Dichanthelium | acuminatum | Pa | 7 | 9 | 0 |  |
| Capillipedium | spicigerum | Pa |  | sig | 1 | Bromus | carinatus | Po | 55 | 48 | 0 |  |
| Aristida | holathera | Ar | 10 | 30 | 1 | Austrostipa | scabra | Po | 29 | 31 | 0 |  |
| Digitaria | breviglumis | Pa |  | sig | 1 | Notodanthonia | racemosa | Da | 70 | 60 | 0 |  |
| Eragrostis | brownii | Ch |  | sig | 1 | Notodanthonia | richardsonii | Da | 100 | 100 | 0 |  |
| Panicum | simile | Pa |  | sig | 1 | Microlaena | stipoides | Eh | 100 | 100 | 0 |  |
| Digitaria | brownii | Pa | 5 | 15/sig | 0.5 | Poa | sieberiana | Po | 94 | 99 | 0 |  |
| Aristida | junciformis | Ar | 85 | 65/sig | 0.5 | Austrostipa | sp | Po |  | NS | 0 |  |
| Themeda | triandra | Pa | 42 | 67/NS | 0.5 | Danthonia | eriantha | Da | 94 | 96 | 0 |  |
| Tristachya | leucothrix | Pa | 30 | 62/NS | 0.5 | Danthonia | linkii var.fulva | Da | 93 | 87 | 0 |  |
| Panicum | mindanaense | Pa | 2 | 6 | 0 | Danthonia | pallida | Da | 53 | 61 | 0 |  |
| Sorghum | intrans | Pa | 6 | 11.5 | 0 | Danthonia | racemosa | Da | 97 | 98 | 0 |  |
| Mnesithea | formosa | Pa | 2 | 0.4 | 0 | Microlaena | stipoides | Eh | 86 | 80 | 0 |  |
| Sorghum | plumosum | Pa | 36 | 20 | 0 | Holcus | lanatus | Po | 100 | 100 | 0 |  |
| Chrysopogon | latifolius | Pa | 34 | 34 | 0 | Bromus | diandrus | Po | 85 | 82 | 0 |  |
| Eriachne | agrostidea | Mi | 30 | 28 | 0 | Festuca | hallii | Po | 63 | 64 | 0 |  |
| Alloteropsis | semialata | Pa | 25 | 20 | 0 | Hesperostipa | comata | Po | 20 | 25 | 0 |  |
| Heteropogon | triticeus | Pa | 24 | 18 | 0 | Nassella | veridula | Po | 15 | 20 | 0 |  |
| Eriachne | avenacea | Mi | 20 | 14 | 0 | Festuca | campestris | Po | 81 | 86 | 0 |  |
| Andropogon | gayanus | Pa | 25 | 28 | 0 | Festuca | frollii | Po | 92 | 94 | 0 |  |
| Andropogon | ascinodis | Pa | 48 | 51 | 0 | Stipa | curtisata | Po | 29 | 34 | 0 |  |
| Diheteropogon | amplectens | Pa | 28 | 29 | 0 | Calemagrostis | canadensis | Po | 26 | 25 | 0 |  |
| Rottboellia | exaltata | Pa | 44.5 | 49 | 0 | Taeniatherum | caput-medusae | Po | 85 | 85 | 0 |  |
| Chasmopodium | caudatum | Pa | 44 | 46 | 0 | Elymus | elymoides | Po | 90 | 92 | 0 |  |
| Cymbopogon | sp. | Pa | 92 | 96 | 0 | Poa | secunda | Po | 30 | 30 | 0 |  |
| *Aristida* | setifolia | Ar | 54 | 64 | 0 | Elymus | cinereus | Po | 15 | 10 | 0 |  |
| *Axonopus* | pressus | Pa | 26 | 32 | 0 | Agrostis | scabra | Po | 99 | 98 | 0 |  |
| *Gymnopogon* | foliosus | Ch | 64 | 58 | 0 | Danthonia | decumbens | Da |  | NS | 0 |  |
| Aristida | riparia | Ar | 95 | 93 | 0 | Festuca | idahoensis | Po |  | NS | 0 |  |
| Aristida | recurvata | Ar | 28 | 39 | 0 | Holcus | lanatus | Po |  | NS | 0 |  |
| Sporobolus | aeneus | Ch | 33 | 37 | 0 | Vulpia | bromoides | Po |  | NS | 0 |  |
| Digitaria | lehmanniana | Pa | 5 | 8 | 0 | Briza | media | Po |  | NS | 0 |  |
| Axonopus | siccus | Pa | 18 | 17 | 0 | Bromus | sterilis | Po |  | NS | 0 |  |
| Eragrostis | curvula | Ch | 68 | 70 | 0 | Bromus | tectorum | Po |  | NS | 0 |  |
| Digitaria | eriantha | Pa |  | NS | 0 | Dichelachne | rara | Po |  | NS | 0 |  |
| Diheteropogon | amplectens | Pa |  | NS | 0 | Entolasia | stricta | Pa |  | NS | 0 |  |
| Eragrostis | plana | Ch |  | NS | 0 | Rytidosperma | racemosum | Da |  | NS | 0 |  |
| Hyparrhenia | hirta | Pa |  | NS | 0 | Rytidosperma | richardsonii | Da |  | NS | 0 |  |
| Paspalum | notatum | Pa |  | NS | 0 | Festuca | valesiaca | Po | 99 | 97 | 0 |  |
| Cynodon | dactylon | Ch |  | NS | 0 | Poa densa | | Po | 49.5 | 37.5 | 0 |  |
| Pennisetum | clandestinum | Pa |  | NS | 0 | Stipa | zalesskii | Po | 3 | 4 | 0 |  |
| Heteropogon | contortus | Pa |  | NS | 0 | Festuca | idahoensis | Po | 70 | 50 | -1 |  |
| Eragrostis | capensis | Ch |  | NS | 0 | Pseudoroegneria | spicata | Po | 30 | 5 | -1 |  |
| Sporobolus | sp. | Ch |  | NS | 0 | Miscanthus | sinensis | Pa | 95 | 76 | -1 |  |
| Microchloa | caffra | Ch |  | NS | 0 | Calamagrostis | hakonensis | Po | 70 | 34 | -1 |  |
| Eragrostis | racemosa | Ch |  | NS | 0 | Stipagrostis | tectorum | Ar | sig |  | -1 |  |
| Eragrostis | dielsii | Ch | 0 | 2 | 0 | Poa | bulbosa | Po | 63 | 20 | -1 |  |
| Eragrostis | eriopoda | Ch | 0 | 0 | 0 | Stipa | scabra | Po | 30 | 20 | -1 |  |
| Eragrostis | tenella | Ch | 1 | 8 | 0 | Hesperostipa | comaa | Po | 50 | 40 | -1 |  |
| Paraneurachne | muelleri | Pa | 10 | 13 | 0 |  |  |  |  |  |  |  |
| Brachyachne | convergens | Ch | 21 | 26 | 0 |  |  |  |  |  |  |  |
| Enneapogon | polyphylla | Pa | 23 | 21 | 0 |  |  |  |  |  |  |  |
| Aristida | latifolia | Ar | 74 | 76 | 0 |  |  |  |  |  |  |  |
| Enneapogon | robustissimus | Pa | 78 | 85 | 0 |  |  |  |  |  |  |  |
| Cymbopogon | ambiguus | Pa | 89 | 94 | 0 |  |  |  |  |  |  |  |
| Cymbopogon | obtectus | Pa | 94 | 95 | 0 |  |  |  |  |  |  |  |
| Aristida | inequiglumis | Ar | 97 | 95 | 0 |  |  |  |  |  |  |  |
| Andropogon | sp. | Pa |  | NS | 0 |  |  |  |  |  |  |  |
| Chasmanthium | sp. | Pa |  | NS | 0 |  |  |  |  |  |  |  |
| Bothriochloa | macra | Pa | 80 | 89 | 0 |  |  |  |  |  |  |  |
| Schizachyrium | sp. | Pa |  | NS | 0 |  |  |  |  |  |  |  |
| Bouteloua | curtipendula | Ch | 83 | 82 | 0 |  |  |  |  |  |  |  |
| Bouteloua | eriopoda | Ch | 16 | 12 | 0 |  |  |  |  |  |  |  |
| Digitaria | ciliaris | Pa | 79 | 76 | 0 |  |  |  |  |  |  |  |
| Panicum | virgatum | Pa | 70 | 70 | 0 |  |  |  |  |  |  |  |
| Dichanthium | setosum | Pa | 97 | 98 | 0 |  |  |  |  |  |  |  |
| Themeda | triandra | Pa | 36 | 42 | 0 |  |  |  |  |  |  |  |
| Bothriochloa | decipiens | Pa | 93 | 94 | 0 |  |  |  |  |  |  |  |
| Chloris | truncata | Ch | 87 | 91 | 0 |  |  |  |  |  |  |  |
| Cymbopogon | refractus | Pa | 44 | 50 | 0 |  |  |  |  |  |  |  |
| Digitaria | brownii | Pa | 18 | 18 | 0 |  |  |  |  |  |  |  |
| Eragrostis | elongata | Ch | 42 | 40 | 0 |  |  |  |  |  |  |  |
| Eriochloa | pseudoacrotricha | Pa | 69 | 68 | 0 |  |  |  |  |  |  |  |
| Echinochloa | crus-galli | Pa |  | NS | 0 |  |  |  |  |  |  |  |
| Aristida | ingrata | Ar | 66 | 50 | 0 |  |  |  |  |  |  |  |
| Chloris | ventricosa | Ch | 90 | 97 | 0 |  |  |  |  |  |  |  |
| Dichanthium | sericeum | Pa | 93 | 98 | 0 |  |  |  |  |  |  |  |
| Aristida | contorta | Ar | 41 | 10 | -1 |  |  |  |  |  |  |  |
| Pseudopogonatherum | contortum | Pa | 39 | 7 | -1 |  |  |  |  |  |  |  |
| Eriachne | triseta | Mi | 34 | 19 | -1 |  |  |  |  |  |  |  |
| Enneapogon | caerulescens | Pa | 52 | 5 | -1 |  |  |  |  |  |  |  |
| Aristida | ramosa | Ar | 94 | 73 | -1 |  |  |  |  |  |  |  |
| Imperata | cylindrica | Pa | sig |  | -1 |  |  |  |  |  |  |  |
| ~~Sporobolus~~ | ~~mitchellii~~ | ~~Ch~~ | ~~15~~ | ~~2~~ | ~~-1~~ |  |  |  |  |  |  |  |

**~~References (for smoke responses, C3-C4 unreferenced as freely available on the web on a per species basis, numbers in square brackets refer to Table 1)~~**

~~Abedi, M., Ezaki, E., Erfanzadeh, R., Naqinezhad, A. 2018. Germination patterns of the scrublands in response to smoke: The role of functional groups and the effect of smoke treatment method. S. Afr. J. Bot. 115, 231–236.~~

~~Abu, Y., Romo, J.T., Bai, Y., Coulman, B. 2016. Priming seeds in aqueous smoke solutions to improve seed germination and biomass production of perennial forage species. Can. J. Plant Sci. 96, 551–563. [19]~~

~~Adkins, S.W., Peters, N.C.B. 2001. Smoke derived from burnt vegetation stimulates germination of arable weeds. Seed Sci. Res. 11, 213–22. [23]~~

~~Blank, R.R., Young, J.A. 1998. Heated substrate and smoke: influence on seed emergence and plant growth. Rangeland Ecol. Manage. 51, 577–83. [22]~~

~~Carthey, A.J., Tims, A., Geedicke, I., Leishman, M.R. 2018. Broad‐scale patterns in smoke‐responsive germination from the SE Australian flora. J. Veg. Sci. 29, 737–745. [17]~~

~~Chou, Y-F., Cox, R.D., Wester, D.B. 2012. Smoke water and heat shock influence germination of shortgrass prairie species. Rangeland Ecol. Manage. 65, 260–267. [9]~~

~~Clarke, P.J., Davison, E.A. Fulloon, L. 2000. Germination and dormancy of grassy woodland and forest species: effects of smoke, heat, darkness and cold. Aust. J. Bot. 48, 687–699. [14]~~

~~Dayamba, S.D., Sawadogo, L., Tigabu., M. et al. 2010. Effects of aqueous smoke solutions and heat on seed germination of herbaceous species of the Sudanian savanna-woodland in Burkina Faso. Flora 205, 319–325. [4]~~

~~Dayamba, S.D., Tigabu, M., Sawadogo, J., Oden, P.C. 2008. Seed germination of herbaceous and woody species of the Sudanian savanna-woodland in response to heat shock and smoke. Forest Ecol. Manage. 256, 462–70. [3]~~

~~Dixon K.W., Roche, S., Pate, J.S. 1995. The promotive effect of smoke derived from burnt native vegetation on seed germination of Western Australian plants.~~ *~~Oecologia~~* ~~101, 185–92. [30]~~

~~Ely, C. 2016. Smoking grass: germination responses of six native Poaceae species to smoke water treatments. Masters Thesis, Evergreen State College, Washington, USA. [21]~~

~~Enright, N.J., Kintrup, A. 2001. Effects of smoke, heat and charred wood on the germination of dormant soil-stored seeds from a~~ *~~Eucalyptus baxteri~~* ~~heathy-woodland in Victoria, SE Australia. Austral Ecol 26, 132–41. [24]~~

~~Erickson, T.E. 2015. Seed dormancy and germination traits of 89 arid zone species targeted for mine-site restoration in the Pilbara region of Western Australia. PhD Thesis, University of Western Australia, Perth, Australia [5]~~

~~Ghebrehiwot, H.M., Kulkarni, M.G., Kirkman K., Van Staden, J. 2012. Smoke and heat: influence on seedling emergence from the germinable soil seed bank of mesic grassland in South Africa. Plant Growth Regul~~*~~.~~* ~~66, 119–127. [6]~~

~~Ghebrehiwot, H.M., Kulkarni, M.G., Kirkman, K.P., Van Staden, J. 2009. Smoke solutions and temperature influence the germination and seedling growth of South African mesic grassland species. Rangeland Ecol Manage. 62, 572–578. [7]~~

~~Gorgone-Barbosa, E., Daibes, L.F., Novaes, R.B., et al~~*~~.~~* ~~2020. Fire cues and germination of invasive and native grasses in the Cerrado. Acta Bot. Bras. 34, 185–91. [12]~~

~~Jefferson, L.V., Pennacchio, M., Havens, K., Forsberg, B., Sollenberger, D., Ault, J. 2008. Ex situ germination responses of Midwestern USA prairie species to plant-derived smoke. Amer. Midland Natural. 159, 251–256. [10]~~

~~Long, R.L., Stevens, J.C., Griffiths, E.M., et al. 2011. Detecting karrikinolide responses in seeds of the Poaceae. Aust. J. Bot. 59, 609–19. [25]~~

~~Naghipour, A.A., Bashari, H., Khajeddin, S.J., et al. 2016. Effects of smoke, ash and heat shock on seed germination of seven species from Central Zagros rangelands in the semi-arid region of Iran. Afr. J. Range Forage Sci. 33, 67–71.~~

~~Osborne, C.P. 2008. Atmosphere, ecology and evolution: what drove the Miocene expansion of C~~_~~4~~_ ~~grasslands? J. Ecol~~*~~.~~* ~~96, 35–45.~~

~~Overbeck G.E., Müller, S.C., Pillar, V.D., Pfadenhauer, J. 2005. No heat-stimulated germination found in herbaceous species from burned subtropical grassland. Plant Ecol. 184, 237–243.~~

~~Paula, S., Pausas, J.G. 2011. Root traits explain different foraging strategies between resprouting life histories. Oecologia 165, 321–331.~~

~~Pausas, J.G., Lamont, B.B. (2022). Fire-released seed dormancy – a global synthesis.~~ *~~Biol. Rev.~~* ~~(in press)~~

~~Penman, T.D., Binns, D., Allen, R., Shiels, R., Plummer, S. 2008. Germination responses of a dry sclerophyll forest soil-stored seedbank to fire related cues. Cunninghamia 10, 547–555.~~

~~Pérez-Fernández, M.A., Rodríguez-Echeverría, S. 2003. Effect of smoke, charred wood, and nitrogenous compounds on seed germination of ten species from woodland in Central-Western Spain. J. Chem. Ecol~~*~~.~~* ~~29, 237–51. [26]~~

~~Ramos, D.M., Valls, J.F.M., Borghetti, F., Ooi, M.K.J. 2019. Fire cues trigger germination and stimulate seedling growth of grass species from Brazilian savannas. Amer. J. Bot. 106, 1190–1201. [13]~~

~~Read, T.R., Bellairs, S.M. 1999. Smoke affects the Germination of Native Grasses of New South Wales. Aust. J. Bot. 47, 563–76. [15]~~

~~Reyes, O., Trabaud, L. 2009. Germination behaviour of 14 Mediterranean species in relation to fire factors: smoke and heat. Plant Ecol. 202, 113–121. [27]~~

~~Roche, S., Dixon, K.W., Pate, J.S. 1998. For everything a season: Smoke-induced seed germination and seedling recruitment in a Western Australian Banksia woodland. Aust. J. Ecol. 23, 111–120. [32]~~

~~Roche, S., Koch, J.W., Dixon, K.W. 1997 Smoke enhanced seed germination for mine rehabilitation in the southwest of Western Australia. Restor. Ecol. 5,191–203. [31]~~

~~Schwilk, D.W., Zavala, N. 2012. Germination response of grassland species to plant-derived smoke. J. Arid. Environ. 79, 111–115. [11]~~

~~Scott K, S Setterfield, M Douglas and A Andersen 2010 Soil seed banks confer resilience to savanna grass-layer plants during seasonal disturbance.~~ *~~Acta Oecol~~* ~~36:202–210. [1]~~

~~Smith, M.A., Bell, D.T., Loneragan, W.A. 1999. Comparative seed germination ecology of~~ *~~Austrostipa compressa~~* ~~and~~ *~~Ehrharta calycina~~* ~~(Poaceae) in a Western Australian Banksia woodland. Aust. J. Ecol. 24, 35–42. [8]~~

~~Stevens, J.C., Merritt, D.J., Flematti, G.R., et al. 2007. Seed germination of agricultural weeds is promoted by the butenolide 3-methyl-2H-furo[2,3-c]pyran-2-one under laboratory and field conditions. Plant Soil 298, 113–124. [28]~~

~~Tang, Y., Boulter, S.L., Kitching, R.L. 2003.Heat and smoke effects on the germination of seeds from soil seed banks across forest edges between subtropical rainforest and eucalypt forest at Lamington National Park, south-eastern Queensland, Australia. Aust. J. Bot. 51, 227–237. [18]~~

~~Tsuyuzaki, S., Miyoshi, C. 2009. Effects of smoke, heat, darkness and cold stratification on seed germination of 40 species in a cool temperate zone in northern Japan. Plant Biol. 11, 369–78. [34]~~

~~Williams, P.R., Congdon, R.A., Grice, A.C., Clarke, P.J. 2005. Germinable soil seed banks in a tropical savanna: seasonal dynamics and effects of fire. Austral Ecol. 30, 79–90. [2]~~

~~Yao, L., Naeth, M.A., Mollard, F.P.O. 2017. Ecological role of pyrolysis by-products in seed germination of grass species. Ecol. Engin. 108, 78–82. [20]~~

~~Zaki, E., Abedi, M., Naqinezhad, A. 2021. How fire history affects germination cues of three perennial grasses from the mountain steppes of Golestan National Park. Flora 280, 151835.~~
